# Proceedings of the First International Conference on Stepped Wedge Trial Design: York, UK, 10 March 2016

**DOI:** 10.1186/s13063-016-1436-8

**Published:** 2016-07-18

**Authors:** Mona Kanaan, Noreen Dadirai Mdege, Ada Keding, RA Parker, N. Mills, A. Shah, F. Strachan, C. Keerie, C. J. Weir, Andrew Forbes, Karla Hemming, Sarah A. Lawton, Emma Healey, Martyn Lewis, Elaine Nicholls, Clare Jinks, Valerie Tan, Andrew Finney, Christian D. Mallen, on behalf of the ENHANCE Study Team, Erik Lenguerrand, Graeme MacLennan, John Norrie, Siladitya Bhattacharya, Tim Draycott, on behalf of the Thistle group, Richard Hooper, Steven Teerenstra, Esther de Hoop, Sandra Eldridge, Alan Girling, Monica Taljaard, Gian Luca Di Tanna, Antonio Gasparrini, Anna Casula, Fergus Caskey, Erik Lenguerrand, Shona Methven, Stephanie MacNeill, Margaret May, Nicholas Selby, Leon Danon, Hannah Christensen, Adam Finn, Margaret May, Fumihito Takanashi, Ada Keding, Simon Crouch, Mona Kanaan, Caroline A. Kristunas, Karen L. Smith, Laura J. Gray, John N.S. Matthews, R. Al-Shahi Salman, R. A. Parker, A. Maxwell, M. Dennis, A. Rudd, C. J. Weir, Jennifer A. Thompson, Katherine L. Fielding, Calum Davey, Alexander M. Aiken, James R. Hargreaves, Richard J. Hayes, Vivian H. Lyons, Lingyu Li, James Hughes, Ali Rowhani-Rahbar, Karla Hemming, Monica Taljaard, Andrew Forbes

**Affiliations:** 1 Department of Health Sciences, University of York, York, North Yorkshire YO10 5DD UK; 2 Usher Institute of Population Health Sciences and Informatics, The University of Edinburgh, Medical School, Teviot Place, Edinburgh, EH8 9AG UK; 3 British Heart Foundation Centre for Cardiovascular Sciences, Chancellors Building, The University of Edinburgh, Edinburgh, EH16 4SB UK; 4 School of Public Health and Preventive Medicine, Monash University, Melbourne, Melbourne, Australia; 5 Department of Public Health, Epidemiology and Biostatistics, University of Birmingham, Birmingham, UK; 6 Research Institute for Primary Care & Health Sciences, Keele University, Keele, Staffs UK; 7 School of Clinical Sciences, University of Bristol, Bristol, BS105NB UK; 8 Centre for Healthcare Randomised Trials, University of Aberdeen, Aberdeen, AB252ZD UK; 9 Applied Health Sciences School of Medicine and Dentistry, University of Aberdeen, Aberdeen, AB252ZD UK; 10 School of Social and Community Medicine, University of Bristol, Bristol, BS105NB UK; 11 Centre for Primary Care & Public Health, Queen Mary University of London, London, UK; 12 Radboud Institute for Health Sciences, Radboud University Medical Centre, Nijmegen, Netherlands; 13 Julius Center for Health Sciences and Primary Care, University Medical Centre Utrecht, Utrecht, Netherlands; 14 Institute of Applied Health Research, University of Birmingham, Birmingham, B15 2TT UK; 15 School of Epidemiology, Public Health, and Preventive Medicine, University of Ottawa, Ottawa, Ontario K1H 8M5 Canada; 16 London School of Hygiene and Tropical Medicine, London, WC1E 7HT UK; 17 UK Renal Registry, Bristol, BS10 5NB UK; 18 School of Social and Community Medicine, University of Bristol, Bristol, BS8 2PS UK; 19 School of Clinical Sciences, University of Bristol, Bristol, BS10 5NB UK; 20 Faculty of Medicine and Health Sciences, University of Nottingham, Nottingham, NG7 2UH UK; 21 Royal Derby Hospital, Derby, DE22 3DT UK; 22 School of Social and Community Medicine, University of Bristol, Bristol, BS8 2BN UK; 23 School of Cellular and Molecular Medicine, University of Bristol, Bristol, BS2 8AE UK; 24 Department of Health Sciences, University of York, York, North Yorkshire YO10 5DD UK; 25 Diabetes Research Centre, University of Leicester, Leicester, UK; 26 Department of Health Sciences, University of Leicester, Leicester, UK; 27 School of Mathematics & Statistics, Newcastle University, Newcastle upon Tyne, NE1 7RU UK; 28 Centre for Clinical Brian Sciences, The University of Edinburgh, Edinburgh, UK; 29 Usher Institute of Population Health Sciences and Informatics, The University of Edinburgh, Medical School, Teviot Place, Edinburgh, EH8 9AG UK; 30 Sentinel Stroke National Audit Programme, Royal College of Physicians, London, UK; 31 Department of Infectious Disease Epidemiology, London School of Hygiene and Tropical Medicine, London, UK; 32 London Hub for Trials Methodology Research, MRC Clinical Trials Unit at University College London, London, UK; 33 Department of Social and Environmental Health Research, London School of Hygiene and Tropical Medicine, London, UK; 34 Department of Epidemiology, University of Washington, Seattle, WA USA; 35 Harborview Injury Prevention & Research Center, Seattle, WA USA; 36 Department of Biostatistics, University of Washington, Seattle, WA USA; 37 School of Health and Population Sciences, University of Birmingham, Birmingham, B15 2TT UK; 38 Clinical Epidemiology Program, Ottawa Hospital Research Institute, 1053 Carling Avenue, Ottawa, Ontario K1Y4E9 Canada; 39 Department of Epidemiology and Community Medicine, University of Ottawa, Ottawa, Ontario Canada; 40 School of Public Health and Preventive Medicine, Monash University, Melbourne, Australia

## Abstract

I1 Introduction

Mona Kanaan, Noreen Dadirai Mdege, Ada Keding

O1 The HiSTORIC trial: a hybrid before-and-after and stepped wedge design

RA Parker, N Mills, A Shah, F Strachan, C Keerie, CJ Weir

O2 Stepped wedge trials with non-uniform correlation structure

Andrew Forbes, Karla Hemming

O3 Challenges and solutions for the operationalisation of the ENHANCE study: a pilot stepped wedge trial within a general practice setting

Sarah A Lawton, Emma Healey, Martyn Lewis, Elaine Nicholls, Clare Jinks, Valerie Tan, Andrew Finney, Christian D Mallen, on behalf of the ENHANCE Study Team

O4 Early lessons from the implementation of a stepped wedge trial design investigating the effectiveness of a training intervention in busy health care settings: the Thistle study

Erik Lenguerrand, Graeme MacLennan, John Norrie, Siladitya Bhattacharya, Tim Draycott, on behalf of the Thistle group

O5 Sample size calculation for longitudinal cluster randomised trials: a unified framework for closed cohort and repeated cross-section designs

Richard Hooper, Steven Teerenstra, Esther de Hoop, Sandra Eldridge

O6 Restricted randomisation schemes for stepped-wedge studies with a cluster-level covariate

Alan Girling, Monica Taljaard

O7 A flexible modelling of the time trend for the analysis of stepped wedge trials: results of a simulation study

Gian Luca Di Tanna, Antonio Gasparrini

P1 Tackling acute kidney injury – a UK stepped wedge clinical trial of hospital-level quality improvement interventions

Anna Casula, Fergus Caskey, Erik Lenguerrand, Shona Methven, Stephanie MacNeill, Margaret May, Nicholas Selby

P2 Sample size considerations for quantifying secondary bacterial transmission in a stepped wedge trial of influenza vaccine

Leon Danon, Hannah Christensen, Adam Finn, Margaret May

P3 Sample size calculation for time-to-event data in stepped wedge cluster randomised trials

Fumihito Takanashi, Ada Keding, Simon Crouch, Mona Kanaan

P4 Sample size calculations for stepped-wedge cluster randomised trials with unequal cluster sizes

Caroline A. Kristunas, Karen L. Smith, Laura J. Gray

P5 The design of stepped wedge trials with unequal cluster sizes

John N.S. Matthews

P6 Promoting Recruitment using Information Management Efficiently (PRIME): a stepped wedge SWAT (study-within-a-trial)

R Al-Shahi Salman, RA Parker, A Maxwell, M Dennis, A Rudd, CJ Weir

P7 Implications of misspecified mixed effect models in stepped wedge trial analysis: how wrong can it be?

Jennifer A Thompson, Katherine L Fielding, Calum Davey, Alexander M Aiken, James R Hargreaves, Richard J Hayes

S1 Stepped Wedge Designs with Multiple Interventions

Vivian H Lyons, Lingyu Li, James Hughes, Ali Rowhani-Rahbar

S2 Analysis of the cross-sectional stepped wedge cluster randomised trial

Karla Hemming, Monica Taljaard, Andrew Forbes

## I1 Introduction

### Mona Kanaan, Noreen Dadirai Mdege, Ada Keding

#### Department of Health Sciences, University of York, York, North Yorkshire, YO10 5DD, UK

##### **Correspondence:** Mona Kanaan – Department of Health Sciences, University of York, York, North Yorkshire, YO10 5DD, UK

The First International Conference on the Stepped Wedge Trial Design was hosted by the York Trials Unit, Department of Health Sciences, University of York on 10 March 2016. The conference brought together national and international delegates with experience, expertise or interest in this design which employs the incremental randomised implementation of an intervention. The design has gained popularity in the health, social and environmental sciences as a tool to enable the evaluation of interventions/policies whilst being rolled out gradually over time.

The conference speakers included eminent researchers at the forefront of the development of the design: Professor Richard Lilford (University of Warwick), Professor Jim Hughes (University of Washington, USA), Dr Karla Hemming (University of Birmingham), Mr. Alan Girling (University of Birmingham), and Professor Andrew Forbes (Monash University, Australia).

The keynote speaker, Professor Richard Lilford opened the conference by giving the context for classic stepped wedge designs. This was followed by nine oral and eight poster presentations where practitioners, researchers and methodologists shared ideas, best practice and challenges for the design, implementation and analysis of the stepped wedge model. The conference was simultaneously broadcast to remotely attending delegates from the UK and abroad via dedicated online channels.

This supplement is a collection of the proceedings of the conference.

## O1 The HiSTORIC trial: a hybrid before-and-after and stepped wedge design

### RA Parker^1^, N Mills^2^, A Shah^2^, F Strachan^1^, C Keerie^1^, CJ Weir^1^

#### ^1^Usher Institute of Population Health Sciences and Informatics, The University of Edinburgh, Medical School, Teviot Place, Edinburgh, EH8 9AG, United Kingdom; ^2^British Heart Foundation Centre for Cardiovascular Sciences, Chancellors Building, The University of Edinburgh, Edinburgh, EH16 4SB, United Kingdom

##### **Correspondence:** RA Parker (richard.parker@ed.ac.uk) – Usher Institute of Population Health Sciences and Informatics, The University of Edinburgh, Medical School, Teviot Place, Edinburgh, EH8 9AG, United Kingdom

**Background**

In six hospitals across Scotland, we are undertaking a study to evaluate the efficacy and safety of implementation of an early rule out diagnostic pathway using a high-sensitivity cardiac troponin to rule out myocardial infarction on presentation in approximately 35,000 consecutive patients with suspected acute coronary syndrome.

**Method**

There will be three study phases each of six months duration: a validation phase using only the standard care pathway; a randomization phase within which the six sites are randomized to start the intervention at one of three time points 8 weeks apart; and an implementation phase calendar matched to the validation phase with all sites using the new pathway. Patients with suspected myocardial infarction will be recruited as they present and then followed-up for 30 days. Sequential hypothesis testing will evaluate two co-primary endpoints in an *a-priori* defined hierarchical order: (1) the proportion of patients discharged from the Emergency Department (efficacy endpoint), and (2) the proportion with myocardial infarction or death at 30 days (safety endpoint test for non-inferiority).

**Results**

The trial is in progress and results are expected in 2018. To control the overall Family-Wise Error Rate for the study we will use a serial gatekeeping procedure.

**Conclusions**

The study design consists of a hybrid before-and-after and stepped wedge trial design. The stepped wedge component enables us to make cross-sectional comparisons as well as within-site comparisons; the before-and-after component allows us to completely adjust for seasonal effects and evaluate the intervention when it is fully embedded into normal practice.

**Acknowledgements**

This abstract is being presented on behalf of the HiSTORIC trial group. The HiSTORIC trial is funded by the British Heart Foundation (PG/15/51/31596). RP and CW were supported in this work by NHS Lothian via the Edinburgh Health Services Research Unit.

## O2 Stepped wedge trials with non-uniform correlation structure

### Andrew Forbes^1^, Karla Hemming^2^

#### ^1^School of Public Health and Preventive Medicine, Monash University, Melbourne, Melbourne, Australia; ^2^Department of Public Health, Epidemiology and Biostatistics, University of Birmingham, Birmingham, UK

##### **Correspondence:** Andrew Forbes (Andrew.Forbes@monash.edu) – School of Public Health and Preventive Medicine, Monash University, Melbourne, Melbourne, Australia

**Background**

Stepped wedge trials have been used with increasing frequency in health research.

For the cross-sectional form of this design, the within-cluster correlation is typically accommodated in the analysis using a random intercept linear mixed model, implying a constant correlation between measurements of any two individuals in the same cluster no matter how far apart in time they are measured.

**Methods**

In this talk we propose an alternate correlation structure in which the within-cluster correlation is allowed to vary depending on the distance between measurements of individuals. In the special case of exponential decay in the within-cluster correlation and an equal number of subjects per period in each cluster, we present results for the variance of treatment effect estimators for varying amounts of decay addressing the following two questions:

- How does the precision in stepped wedge trials compare to parallel-group cluster trials of the same size as the decay varies?
- What are the consequences of this variation for sample size planning?

**Results and conclusions**

Our results indicate that in certain design configurations a correlation decay can have an impact on the variance of treatment effect estimators, and hence on sample size and power.

## O3 Challenges and solutions for the operationalisation of the ENHANCE study: a pilot stepped wedge trial within a general practice setting

### Sarah A Lawton, Emma Healey, Martyn Lewis, Elaine Nicholls, Clare Jinks, Valerie Tan, Andrew Finney, Christian D Mallen, on behalf of the ENHANCE Study Team

#### Research Institute for Primary Care & Health Sciences, Keele University, Keele, Staffs, UK

##### **Correspondence:** Sarah A Lawton (s.a.lawton@keele.ac.uk) – Research Institute for Primary Care & Health Sciences, Keele University, Keele, Staffs, UK

**Background**

The ENHANCE pilot trial aims to examine the feasibility and acceptability of an integrated approach to Long Term Condition (LTC) management by tackling the under-diagnosis and under-management of osteoarthritis (OA) related pain and anxiety &/or depression in patients aged 45 years and over with other LTCs in primary care, using a stepped wedge trial design. This abstract describes some of the challenges faced in operationalising this trial design within general practice, together with solutions that have been implemented.

**Method**

The intervention is an ‘ENHANCE’ LTC review, delivered by Practice Nurses and in accordance with a stepped wedge design, has been rolled out to four general practices (clusters) over time. Operationalisation challenges linked to the methods required for this trial design have included; Scheduling of intervention training visits to fit with randomisation schedules; Initial recruitment enthusiasm waning prior to implementation of the intervention phase; Increase in trial delivery requirements within clusters as the practice moves into the intervention phase.

**Results**

In order to address these challenges the following solutions have been implemented; a 2 week ‘wash out’ period to ensure clusters make a smooth transition from control to intervention phase; Dedicated trial management communication, forward planning and organisation of intervention training delivery; Identical study materials across control and intervention phases; Communication and updates around recruitment figures to ensure recruitment and adherence to study design.

**Conclusions**

The stepped wedge design is an attractive option for delivering an intervention within complex settings, however presents challenges for implementation which need careful planning.

**Trial registration**

ISRCTN 12154418

## O4 Early lessons from the implementation of a stepped wedge trial design investigating the effectiveness of a training intervention in busy health care settings: the Thistle study

### Erik Lenguerrand^1^, Graeme MacLennan^2^, John Norrie^2^, Siladitya Bhattacharya^3^, Tim Draycott^4^ on behalf of the Thistle group

#### ^1^School of Clinical Sciences, University of Bristol, Bristol, BS105NB, UK; ^2^Centre for Healthcare Randomised Trials, University of Aberdeen, Aberdeen, AB252ZD, UK; ^3^Applied Health Sciences School of Medicine and Dentistry, University of Aberdeen, Aberdeen, AB252ZD; ^4^School of Social and Community Medicine, University of Bristol, Bristol, BS105NB, UK

##### **Correspondence:** Erik Lenguerrand (erik.lenguerrand@bristol.ac.uk) – School of Clinical Sciences, University of Bristol, Bristol, BS105NB, UK

**Background**

There is increasing methodological literature on design, sample size calculations and analyses of stepped wedge trials (SWT). However, the challenges encountered and potential solutions developed during the implementation of SWTs are described less well. We aim to share the experience of implementing the Thistle study, an on-going SWT evaluating the effectiveness of a multi-professional obstetric training programme across a health service.

**Method**

Our 36-month study consists of 12 Scottish maternity units randomised in groups of four, to three intervention-steps of 6-months length. Teams from each unit were trained in how to deliver the intervention. The primary outcome (Apgar score) will be modelled using marginal logistic regression following the intention-to-treat principle (ITT).

**Results**

Departures from the randomisation plan were required to accommodate clinical constraints at four Maternity Units and ensure that they were retained within the study. Heterogeneity in the timing and frequency of local training post implementation was observed; some units started prior to their allocated intervention step, some after, and some completed their implementation over several steps. We will use the wealth of routinely collected clinical and training data to supplement the ITT-analysis with several sensitivity analyses to account for the actual intervention implementation.

**Conclusions**

Using a SWT design to evaluate the effectiveness of training intervention in busy health care settings is complex. We have highlighted problems regarding adherence to allocated step and the sensitivity analyses we propose to tackle them. These findings will help guide investigators in the designing and analysis of future SWTs.

**Trial registration**

UKCRN ID: 15400

## O5 Sample size calculation for longitudinal cluster randomised trials: a unified framework for closed cohort and repeated cross-section designs

### Richard Hooper^1^, Steven Teerenstra^2^, Esther de Hoop^3^, Sandra Eldridge^1^

#### ^1^Centre for Primary Care & Public Health, Queen Mary University of London, London, UK; ^2^Radboud Institute for Health Sciences, Radboud University Medical Centre, Nijmegen, Netherlands; ^3^Julius Center for Health Sciences and Primary Care, University Medical Centre Utrecht, Utrecht, Netherlands

##### **Correspondence:** Richard Hooper (r.l.hooper@qmul.ac.uk) – Centre for Primary Care & Public Health, Queen Mary University of London, London, UK

**Background**

Recent articles have considered stepped wedge trials as part of a broader class of cluster randomised trials where two or more independent cross-sections are taken from each cluster at fixed times, with all participants in any given cross-section in any given cluster receiving either the experimental or the control treatment. A unified approach to sample size calculation has been proposed in such cases. However, a method for calculating sample size for closed cohort cluster randomised trials, in which the same participants from each cluster are followed repeatedly over time, has not yet been described in the literature.

**Methods**

Here we show how common principles apply both to closed cohort and to repeated cross-section cluster randomised trials, allowing a unified framework for sample size calculation.

**Results**

Our general formulae are consistent with those previously described in special cases such as stepped wedge, parallel group, and cross-over designs, as well as being a natural extension of formulae for individually randomised trials with repeated assessments. Our framework is more general than that of Hussey & Hughes in that we include an additional parameter, which we call the cluster autocorrelation, allowing participants from the same cluster sampled at different times to be less well correlated than participants from the same cluster sampled at the same time.

**Discussion**

We discuss the practical importance of the cluster autocorrelation and other nuisance parameters, and the possible limitations of our underlying statistical model. We also consider simulation as a tool for assessing small-sample inaccuracies in asymptotic formulae.

## O6 Restricted randomisation schemes for stepped-wedge studies with a cluster-level covariate

### Alan Girling^1^, Monica Taljaard^2^

#### ^1^Institute of Applied Health Research, University of Birmingham, Birmingham, B15 2TT, UK; ^2^School of Epidemiology, Public Health, and Preventive Medicine, University of Ottawa, Ottawa, Ontario, K1H 8M5, Canada

##### **Correspondence:** Alan Girling (a.j.girling@bham.ac.uk) – Institute of Applied Health Research, University of Birmingham, Birmingham, B15 2TT, UK

**Background**

Stratified randomisation has been recommended to balance the distribution of a cluster-level covariate over the arms of a stepped-wedge study. The strata may reflect a simplified categorisation of the covariate, and the approach is not available in studies with a single cluster in each arm.

**Methods**

We assume that a potential effect-modifier – i.e. a covariate that interacts with the intervention – can be used to generate a prior ordering of the clusters. The size of a health-service unit is a popular candidate here. An “anti-symmetric” randomisation scheme is described in which clusters occupying equally extreme (but opposite) positions in the ordering are assigned either to the same arm of the design, or to the corresponding arm in the opposite half of the design. The procedure is illustrated for some recent and ongoing studies.

**Results**

Under simplified modelling assumptions the resulting designs satisfy two valuable properties of traditional stratified designs: (a) in an unadjusted analysis the estimate of the average treatment effect is unbiased; (b) in an adjusted analysis the precision of the average effect estimate is maximised. Simulation results are presented to compare the bias for anti-symmetric designs with other randomisation schemes.

**Conclusions**

In a stepped-wedge study the potential for cluster-level confounders to interfere with the treatment effect estimate is limited because both treatment conditions occur within every cluster. The impact of a potential effect-modifier can be mitigated using a restricted randomisation scheme. Anti-symmetric randomisation offers advantages over common-sense schemes which seek to balance the effect-modifier over the treatment conditions. Unlike conventional stratification this scheme is available even where no replication of the design is possible.

## O7 A flexible modelling of the time trend for the analysis of stepped wedge trials: results of a simulation study

### Gian Luca Di Tanna, Antonio Gasparrini

#### London School of Hygiene and Tropical Medicine, London, WC1E 7HT, UK

##### **Correspondence:** Gian Luca Di Tanna (Gianluca.ditanna@lshtm.ac.uk) – London School of Hygiene and Tropical Medicine, London, WC1E 7HT, UK

**Background**

For the analysis of SWTs two main approaches have been proposed: the vertical approach, where the intervention and the control groups are compared within periods between successive switching points, conditioning on time and the horizontal approach that takes into account of secular changes by including a fixed-effects indicator for each time point. Here we propose an alternative model for horizontal analyses, where cluster-specific secular trends are more flexibly modelled through linear random terms.

**Methods**

The standard and alternative models are compared in a simulation study. Specifically, we simulated binary and continuous outcomes in a SWT with 20 clusters, adopting different choices regarding the number of steps (5-10) and participants per cluster (25-50). We evaluated 4 different scenarios: 1) stable trend, 2) increasing linear trend, 3) cluster-specific linear trends and 4) cluster-specific trends with completely random patterns.

**Results**

In all scenarios the two models provide unbiased estimates of the effect of the intervention and similar efficiency in terms of root mean square error and power. For SWTs with 10 steps, the alternative model outperforms the standards one, with the latter showing undercoverage in the third scenario, and generally lower convergence rates. Both models suffer from quite low coverage in the fourth, less plausible scenario.

**Conclusions**

Our method represents a valid alternative to the traditional analytical approach for SWTs: while maintaining similar statistical power, our approach shows better inferential and computational properties, provides additional information on cluster-specific trends and can flexibly accommodate non-standard situations such as unequal time measurements across clusters for generic SW designs.

## P1 Tackling acute kidney injury – a UK stepped wedge clinical trial of hospital-level quality improvement interventions

### Anna Casula^1^, Fergus Caskey^1,2^, Erik Lenguerrand^3^, Shona Methven^1,3^, Stephanie MacNeill^1,2^, Margaret May^2^, Nicholas Selby^4,5^

#### ^1^UK Renal Registry, Bristol, BS10 5NB, UK; ^2^School of Social and Community Medicine, University of Bristol, Bristol, BS8 2PS, UK; ^3^School of Clinical Sciences, University of Bristol, Bristol, BS10 5NB, UK; ^4^Faculty of Medicine and Health Sciences, University of Nottingham, Nottingham, NG7 2UH, UK; ^5^Royal Derby Hospital, Derby, DE22 3DT, UK

##### **Correspondence:** Anna Casula (laura.woodward@renalregistry.nhs.uk) – UK Renal Registry, Bristol, BS10 5NB, UK

**Background**

Acute kidney injury (AKI) is a sudden reduction in kidney function frequently observed during hospitalisation and associated with multiple negative outcomes that may be amenable to early intervention. Tackling-AKI, a quality improvement project, aims to assess the effectiveness of a package of hospital-level interventions for AKI, using patient-level outcomes collected pre- and post-intervention within a stepped wedge study design.

**Methods**

All adults hospitalised overnight and sustaining AKI, referred to the five participating UK hospitals, will be included.

The package of interventions comprises:

- An electronic detection system to improve early recognition of AKI
- An education programme to raise staff awareness and knowledge in all major medical and surgical specialities
- A care bundle to improve the delivery of basic components of AKI care

The study is taking place between December’14 and November’16, with steps of three months. There will be two control periods, five intervention steps with one hospital randomised to each step, and at least one final follow-up step. Primary outcome is 30-day mortality after AKI. Assuming an average of 540 AKI episodes per hospital per time-period, 16 % 30-day mortality, α = 0.05 and ICC = 0.01, we will have 80 % power to detect 20 % decrease in mortality.

**Results**

By February 2016, three of the five hospitals should have implemented the intervention. Trial design and protocol will be described, including barriers in data collection and adherence to protocol.

**Conclusions**

This trial aims to test the effectiveness of the care package and will provide evidence to determine the relevance of upscaling at national UK level.

**Acknowledgements**

This trial is supported by the Health Foundation.

## P2 Sample size considerations for quantifying secondary bacterial transmission in a stepped wedge trial of influenza vaccine

### Leon Danon^1^, Hannah Christensen^1^, Adam Finn^2^, Margaret May^1^

#### ^1^School of Social and Community Medicine, University of Bristol, Bristol, BS8 2BN, UK; ^2^School of Cellular and Molecular Medicine, University of Bristol, Bristol, BS2 8AE, UK

##### **Correspondence:** Hannah Christensen (hannah.christensen@bristol.ac.uk) – School of Social and Community Medicine, University of Bristol, Bristol, BS8 2BN, UK

**Background**

Quantifying the impact of bacterial density on transmission is important for understanding population level secondary effects of vaccines. We developed a stepped wedge trial (SWT) to understand the effects of Live Attenuated Influenza Vaccination (LAIV) on bacterial transmission by measuring impact of bacterial colonisation density on transmission. We present sample size calculations for this SWT.

**Materials and method**

The design involved giving 2 year-old children LAIV, which transiently increases *S.pneumoniae* (Sp) density in recipients, and measuring bacterial transmission to household contacts. Index children will be randomised to receive LAIV at the first or third of five fortnightly home visits, when microbiological samples will be collected from them and their contacts. Data relating carriage density and transmission, and clustering within families were unavailable, but evidence suggests 60-70 % of pre-school children and >15 % of contacts are Sp carriers. Extrapolation of census data suggested an average of 2.46 contacts per child.

**Results**

Detection of a 2-fold rise in transmission with 90 % power requires a sample of 260 household contacts of children carrying Sp at the outset per study arm (total 520) assuming a normal approximation to the binomial distribution. 500 index children and their families, yielding 1230 contacts would be sufficient for endpoint detection, allowing for 20 % dropout and ≥60 % carriage rate in the index (providing 590 contacts of informative index cases, 500x2.46x0.8x0.6). Social contact data collected to stratify contacts by duration and proximity, will be discussed at conference.

**Conclusion**

The lack of validated methods for sample size calculations of complex SWT presents challenges.

**Acknowledgements**

HC is supported by, and AF is a member of, the National Institute for Health Research Health Protection Research Unit (NIHR HPRU) in Evaluation of Interventions at University of Bristol in partnership with Public Health England (PHE). The views expressed are those of the author(s) and not necessarily those of the NHS, the NIHR, the Department of Health or Public Health England.

## P3 Sample size calculation for time-to-event data in stepped wedge cluster randomised trials

### Fumihito Takanashi, Ada Keding, Simon Crouch, Mona Kanaan

#### Department of Health Sciences, University of York, York, North Yorkshire, YO10 5DD, UK

##### **Correspondence:** Mona Kanaan (mona.kanaan@york.ac.uk) – Department of Health Sciences, University of York, York, North Yorkshire, YO10 5DD, UK

**Background**

Stepped wedge cluster randomised trial (SWCRT) designs are increasingly popular for the evaluation of health interventions. However, methodologies for appropriate sample size calculation have been limited to specific design scenarios [1, 2], namely, continuous and binary outcomes. To our knowledge, no methodology is available yet specifically to time-to-event data. This study aimed to evaluate sample size and statistical power for SWCRTs that measure time-to-event data.

**Methods**

A model of the SWCRT design and time-to-event data was developed and simulated in R [3]. Relevant design parameters were then assessed for their impact on statistical power.

**Results**

Simulations showed that several parameters changed statistical power while others had no observable effect in ways that are normally expected. Furthermore, the methodology was applied to estimate the power of a published SWCRT.

**Conclusions**

We expect the proposed power estimation method to support the efficient planning of future time-to-event SWCRTs. The method is sufficiently flexible to allow further development by incorporating many design issues encountered in real studies such as more expanded models of time-to-event data and various censoring mechanisms.

**References**

1. Hussey MA, Hughes JP: **Design and analysis of stepped wedge cluster randomized trials**. *Contemporary Clinical Trials* 2007; 28(2):182-191.

2. Reich NG, Myers JA, Obeng D, Milstone AM, Perl TM: **Empirical Power and Sample Size Calculations for Cluster-Randomized and Cluster-Randomized Crossover Studies**. *Plos One* 2012; **7**(4):e35564.

3. Bender R, Augustin T, Blettner M: **Generating survival times to simulate Cox proportional hazards models.***Statistics in Medicine* 2005; **24**(11):1713-1723.

## P4 Sample size calculations for stepped-wedge cluster randomised trials with unequal cluster sizes

### Caroline A. Kristunas^1^, Karen L. Smith^2^, Laura J. Gray^2^

#### ^1^Diabetes Research Centre, University of Leicester, Leicester, UK; ^2^Department of Health Sciences, University of Leicester, Leicester, UK

##### **Correspondence:** Caroline A. Kristunas (cak21@le.ac.uk) – Diabetes Research Centre, University of Leicester, Leicester, UK

**Background**

The current methodology for sample size calculation for stepped-wedge cluster randomised trials (SW-CRTs) is based on the assumption of the clusters being of equal size. However, as is often the case in CRTs, the clusters in SW-CRTs are likely to vary in size which in CRTs of other designs leads to a reduction in power. The effect of an imbalance in cluster sizes on SW-CRTs was not known, nor what an appropriate adjustment to the sample size should be.

**Methods**

We proposed three adjusted design effects (DEs) for use in the calculation of the sample size for SW-CRTs with varying degrees of imbalance in cluster size, based on those suggested for use in CRTs with unequal cluster sizes. A simulation study was conducted which investigated the effect of unequal cluster sizes on the power of SW-CRTs, when the sample size was calculated using both the standard method and the three proposed adjusted DEs.

**Results**

An imbalance in cluster size was not found to significantly affect the power of a SW-CRT, and the proposed adjusted DEs generally resulted in trials that were severely over-powered.

**Conclusions**

We recommend that the standard method of sample size calculation for SW-CRTs be used when any imbalance in cluster size is expected to be small. When there is likely to be a large imbalance in cluster size it is recommended that simulations be used to determine if additional clusters are needed.

**Acknowledgements**

CK is funded by a National Institute for Health Research (NIHR) Research Methods Fellowship. The views expressed in this publication are those of the author(s) and not necessarily those of the NHS, the NIHR or the Department of Health.

## P5 The design of stepped wedge trials with unequal cluster sizes

### John N.S. Matthews (john.matthews@ncl.ac.uk)

#### School of Mathematics & Statistics, Newcastle University, Newcastle upon Tyne, NE1 7RU, UK

**Background**

A stepped wedge (SW) design, comparing standard treatment (A) with a new treatment (B), can be thought of as allocating units to *L* sequences of treatments, with each sequence divided into *T* successive periods. In the usual SW design the *L* and *T* are such that *L* = *T*-1. It may be infeasible to run a trial with many treatment periods but at the same time the number of units, *N*, to be allocated will exceed *T*-1, so multiple units are allocated to each sequence. The units are usually clusters, such as general practices, not of equal size. The problem then arises about how best to allocate a set of *N* units, of known sizes, *n*_1_,*n*_2_,…,*n*_*N*_, to the *L* sequences.

**Method**

Assuming the standard model proposed by Hussey & Hughes [1], an expression for the variance, *V*, of the estimator of the treatment effect, is derived using methods from optimal design theory. The optimal allocation of the units to sequences can be found by minimizing the value of *V*.

**Results**

Exact results are available when the intra-class correlation (ICC) is extreme. For more realistic values and modest numbers of clusters (< about 10), the optimal design can be found using exhaustive searches and these suggest a smooth transition between the forms of the design for extreme ICCs. Approaches using suitable approximations are needed for larger numbers of clusters.

**Conclusion**

With SW designs with clusters of varying sizes attention should be paid to how these are allocated to the sequences in the design.

**References**

1. Hussey MA, Hughes JP: **Design and analysis of stepped wedge cluster randomized trials.***Contemp Clin Trials* 2007; **28:** 182-191.

## P6 Promoting Recruitment using Information Management Efficiently (PRIME): a stepped wedge SWAT (study-within-a-trial)

### R Al-Shahi Salman^1^, RA Parker^2^, A Maxwell^1^, M Dennis^1^, A Rudd^3^, CJ Weir^2^

#### ^1^Centre for Clinical Brian Sciences, The University of Edinburgh, Edinburgh, UK; ^2^Usher Institute of Population Health Sciences and Informatics, The University of Edinburgh, Medical School, Teviot Place, Edinburgh, EH8 9AG, UK; ^3^Sentinel Stroke National Audit Programme, Royal College of Physicians, London, UK

##### **Correspondence:** RA Parker (richard.parker@ed.ac.uk) – Usher Institute of Population Health Sciences and Informatics, The University of Edinburgh, Medical School, Teviot Place, Edinburgh, EH8 9AG, UK

**Background**

In general, recruitment is a challenge for trials of secondary prevention after stroke in the UK. Certainly it is an ongoing challenge for the REstart or STop Antithrombotics Randomised Trial (RESTART): a multicentre trial in stroke prevention.

**Method**

We are currently conducting a stepped wedge cluster randomised trial of a complex intervention to boost recruitment at 72 active sites in RESTART. The intervention involves a recruitment co-ordinator who is (1) providing software for hospital sites to extract lists of their own patients from stroke audit data sources using criteria customised to the trial eligibility criteria, (2) training investigators at each site via a telephone ‘recruitment review’ to use the reports and approach prevalent stroke survivors, and (3) following-up the recruitment review 6 months later. The primary outcome of site recruitment rate will be compared before and after implementation of the recruitment reviews in a negative binomial mixed model, adjusting for site, time since start of study, and season.

**Results**

Stratified block randomisation was used to randomly allocate the 72 sites to one of 12 specific timings when they would start to implement the intervention, stratified by hospital location (Scotland versus England & Wales). The trial has been registered in the SWAT repository [www.qub.ac.uk/sites/TheNorthernIrelandNetworkforTrialsMethodologyResearch/SWATSWARInformation/Repositories/SWATStore/].

**Conclusions**

This is an example of a study-within-a-trial with a closed cohort stepped wedge design whereby all sites begin in the control state and the monthly recruitment rate is measured until after all sites have been allocated to the intervention.

**Acknowledgements**

The PRIME trial is funded by the British Heart Foundation (Ref: PG/14/50/30891). RP and CW were supported in this work by NHS Lothian via the Edinburgh Health Services Research Unit. RA-SS was supported by a MRC senior clinical fellowship.

## P7 Implications of misspecified mixed effect models in stepped wedge trial analysis: how wrong can it be?

### Jennifer A Thompson^1,2^, Katherine L Fielding^1^, Calum Davey^3^, Alexander M Aiken^3^, James R Hargreaves^3^, Richard J Hayes^1^

#### ^1^Department of Infectious Disease Epidemiology, London School of Hygiene and Tropical Medicine, London, UK; ^2^ London Hub for Trials Methodology Research, MRC Clinical Trials Unit at University College London, London, UK; ^3^ Department of Social and Environmental Health Research, London School of Hygiene and Tropical Medicine, London, UK

##### **Correspondence:** Jennifer A Thompson (jennifer.thompson@lshtm.ac.uk) – Department of Infectious Disease Epidemiology, London School of Hygiene and Tropical Medicine, London, UK

**Background**

Many stepped wedge trials are analysed using a mixed effect model with a random effect for cluster but there is little understanding of the implications of misspecifying this model. We investigated the estimated intervention effect and its standard error when time period and intervention effects varied between clusters but were treated as fixed effects in the analysis model.

**Methods**

We performed a simulation study of a stepped wedge trial with three groups and two time periods: during the first period one group had the intervention, and during the second two groups had the intervention. We simulated combinations of time period and intervention effects being common to all or varying between clusters. These simulated data were analysed with a mixed effect model with a random effect for cluster only, or with additional random effects for time period or intervention.

**Results**

Omitting random effects for time period or intervention in the analysis model when variation between clusters was present in these effects led to standard errors which were much too small and type 1 error rates of up to 94 %. Estimated intervention effects remained unbiased with all analysis models. Inclusion of a random effect for either time period or intervention effect in the analysis model improved the type 1 error rate when there was variability between clusters of either effect present.

**Conclusions**

Stepped wedge trial analyses must account for variability between clusters in time period and intervention effects in order to appropriately reflect the precision of the intervention effect estimate.

## S1 Stepped Wedge Designs with Multiple Interventions

### Vivian H Lyons^1,2^, Lingyu Li^3^, James Hughes^3^, Ali Rowhani-Rahbar^1,2^

#### ^1^Department of Epidemiology, University of Washington, Seattle, WA, USA; ^2^Harborview Injury Prevention & Research Center, Seattle, WA, USA; ^3^Department of Biostatistics, University of Washington, Seattle, WA, USA

##### **Correspondence:** James Hughes (jphughes@u.washington.edu) – Department of Biostatistics, University of Washington, Seattle, WA, USA

**Background**

Stepped wedge design trials, in which each cluster crosses-over unidirectionally from a control to an intervention condition, are typically used to evaluate a single intervention. We examined variations of stepped wedge designs for evaluating multiple interventions.

**Methods**

We describe four variants of a stepped wedge design trial with two interventions: concurrent design (two single intervention stepped wedge trials implemented simultaneously), replacement (unidirectional cross-over from control to first intervention to second intervention), supplementation (unidirectional cross-over from control to first intervention to combined intervention), and factorial designs (half the clusters cross-over from control to first intervention to combined intervention and half cross-over from control to second intervention to combined intervention). Analyses are conducted comparing the precision of the estimated intervention effects for the different designs.

**Results**

Under the Hussey and Hughes (2007) [1] model for stepped wedge designs, the concurrent, supplementation, and factorial variants provide equal precision for estimating the treatment effects, while in the replacement design the first introduced intervention is generally estimated more precisely than the second intervention. Surprising and nonintuitive changes in the precision of the intervention effect estimates are observed when additional observation time intervals are included in these designs. Results may depend on the model chosen for analysis.

**Conclusion**

These variations offer methods for studying two interventions using a stepped wedge design. Selection of a design should be driven primarily by the research question with additional consideration given to the trade-off between trial duration and number of clusters, as well as restrictions for concurrent or sequential implementation based on intervention characteristics.

[1] Hussey MA, Hughes JP. Design and Analysis of Stepped Wedge Cluster Randomized Trials. *Contemporary Clinical Trials, 28:182-191,* 2007.

[2] Lyons VH, Li L, Hughes JP, Rowhani-Rahbar A. *Evaluation of Multiple Interventions Using a Stepped Wedge Design*. University of Washington Department of Biostatistics Technical Report. Working Paper 411. Seattle, WA; 2016. http://biostats.bepress.com/uwbiostat/paper411

## S2 Analysis of the cross-sectional stepped wedge cluster randomised trial

### Karla Hemming^1^, Monica Taljaard^2,3^, Andrew Forbes^4^

#### ^1^School of Health and Population Sciences, University of Birmingham, Birmingham, B15 2TT UK; ^2^Clinical Epidemiology Program, Ottawa Hospital Research Institute, 1053 Carling Avenue, Ottawa, Ontario, K1Y4E9, Canada; ^3^Department of Epidemiology and Community Medicine, University of Ottawa, Ottawa, Ontario, Canada; ^4^School of Public Health and Preventive Medicine, Monash University, Melbourne, Australia

##### **Correspondence:** Karla Hemming (k.hemming@bham.ac.uk) – School of Health and Population Sciences, University of Birmingham, Birmingham, B15 2TT UK

**Background**

Stepped wedge cluster randomised trials (SW-CRT) are novel study designs increasingly used to evaluate policy or service delivery treatments. There is a dearth of literature on how to analyse these studies. A recent systematic review identified that 67 % of published SW-CRTs failed to adjust for secular trends at the analysis stage.

**Methods**

We set out a framework for how results from cross-sectional SW-CRTs should be analysed. We recommend that as with all cluster trials, allowance should be made for the clustered nature of the data. In addition, adjustment should be made for underlying secular trends, irrespective of whether these are identified as statistically significant. We allow for different secular trends in different cluster stratum; an allowance for treatment effect heterogeneity across clusters; variation in the treatment effect over time since introduction into the cluster; and include an inter-period as well as an inter-cluster correlation.

**Results**

We illustrate these analysis methods using a case study. In this case study the unadjusted effect of the treatment suggests that the treatment is beneficial. However, we demonstrate evidence of an underlying secular trend with the outcome improving in control clusters over time. As a result, after adjustment for secular trends, the adjusted treatment effect reveals no effect of the treatment and may even suggest harm. When allowing for a lag effect, this difference was even more pronounced. Other model variations considered had no substantial impact on conclusions in the example.

**Conclusion**

When interpreting and analysing a SW-CRT the estimated treatment effect should be adjusted for secular trends. This adjusted treatment effect can be very different to the unadjusted treatment effect. Furthermore, the analysis methods are not assumption free and the appropriateness of these assumptions should be investigated.

